# Oncogenic histone methyltransferase EZH2: A novel prognostic marker with therapeutic potential in endometrial cancer

**DOI:** 10.18632/oncotarget.16316

**Published:** 2017-03-17

**Authors:** Shinya Oki, Kenbun Sone, Katsutoshi Oda, Ryuji Hamamoto, Masako Ikemura, Daichi Maeda, Makoto Takeuchi, Michihiro Tanikawa, Mayuyo Mori-Uchino, Kazunori Nagasaka, Aki Miyasaka, Tomoko Kashiyama, Yuji Ikeda, Takahide Arimoto, Hiroyuki Kuramoto, Osamu Wada-Hiraike, Kei Kawana, Masashi Fukayama, Yutaka Osuga, Tomoyuki Fujii

**Affiliations:** ^1^ Department of Obstetrics and Gynecology, Faculty of Medicine, The University of Tokyo, Tokyo 113-8655, Japan; ^2^ Division of Molecular Modification and Cancer Biology, National Cancer Center Research Institute, Tokyo 104-0045, Japan; ^3^ Department of Pathology, Faculty of Medicine, The University of Tokyo, Tokyo 113-8655, Japan; ^4^ Department of Pathology, Faculty of Medicine, Akita University, Akita 010-8543, Japan; ^5^ Center for Female Preventive Medicine, Kanagawa Health Service Association, Naka-ku, Yokohama, Kanagawa 231-0021, Japan

**Keywords:** histone methyltransferase, EZH2, endometrial cancer, GSK126, H3K27 trimethylation

## Abstract

The histone methyltransferase EZH2, a key epigenetic modifier, is known to be associated with human tumorigenesis. However, the physiological importance of EZH2 and its clinical relevance in endometrial cancer remain unclear. Hence, in the present study, we investigated the expression and function of EZH2 in endometrial cancer. In a quantitative real-time PCR analysis of 11 endometrial cancer cell lines and 52 clinical endometrial cancer specimens, EZH2 was significantly overexpressed in cancer cells and tissues compared to that in corresponding normal control cells and tissues. Kaplan-Meier survival analysis using data of the TCGA RNA-seq database and tissue microarrays (TMAs) indicated that EZH2 overexpression is associated with endometrial cancer prognosis. In addition, knockdown of EZH2 using specific siRNAs resulted in growth suppression and apoptosis induction of endometrial cancer cells, accompanied by attenuation of H3K27 trimethylation. Consistent with these results, treatment with GSK126, a specific EZH2 inhibitor, suppressed endometrial cancer cell growth and decreased the number of cancer cell colonies. Furthermore, GSK126 showed additive effects with doxorubicin or cisplatin, which are conventional drugs for treatment of endometrial cancer. Further studies should explore the therapeutic potential of inhibiting EZH2 in patients with endometrial cancer.

## INTRODUCTION

Endometrial cancer is one of the most common gynecological malignancies, the incidence of which is increasing worldwide. This increase is ascribed to the rising prevalence of obesity and nulliparity [1]. Although the prognosis of low-risk endometrial cancer is generally favorable, chemotherapeutic options for high-risk patients are limited, and there are no approved molecular therapies thus far; therefore, the development of novel therapeutic strategies is necessary to improve the prognosis.

Chromosomes within eukaryotic nuclei combine with structural proteins such as histones to form chromatin. Four major histones (H2A, H2B, H3, and H4) form an octamer comprising two copies of each histone type, around which DNA is wound to form regular, repeating units known as nucleosomes [2]. These nuclear histones undergo various chemical modifications such as acetylation, methylation, ubiquitination, sumoylation, poly ADP-ribosylation, and phosphorylation [3]. Among these modifications, dynamic upregulation or downregulation of histone methylation is required for epigenetic modification of gene expression. Many types of histone methyltransferases and demethylases have been identified to have critical roles in methylation. A large body of evidence has indicated that dysregulation of histone methylation is involved in the development and progression of cancer. Several histone methyltransferases and demethylases have been reported to be overexpressed in various types of cancers [4–6]. For example, we reported that the histone methyltransferase SUV39H2 induced cell proliferation and chemo- and radio-resistance in lung cancer cells [7]. Enzymes relevant to histone methylation and involved in human tumorigenesis have recently attracted attention for anti-cancer drug development [6].

Enhancer of zeste homolog 2 (EZH2) is a histone methyltransferase that methylates lysine 27 of histone H3 to promote transcription silencing [8, 9]. EZH2 is the enzymatically active core subunit of the PRC2 complex, which also includes EED, SUZ12, RbAp46, and RbAp48 [10]. EZH2 has been reported to be overexpressed in many types of cancer, and high levels of EZH2 are associated with tumor aggressiveness [11, 12]. Increased EZH2 activity is known to induce H3K27 trimethylation and act as an oncogene by repressing tumor suppressor genes [13]. Accumulating evidence demonstrates the potential of EZH2 expression for the development of anti-cancer therapeutics. Several groups have reported compounds that directly and selectively inhibit EZH2 activity [14–16]. GSK126, an EZH2 inhibitor, markedly suppresses the growth of several types of cancer, including B-cell lymphoma [17].

Although previous studies have examined EZH2 expression in endometrial cancer, there has been no comprehensive analysis of EZH2 expression and function in endometrial cancer using EZH2-selective inhibitors [18–21]. We therefore investigated the involvement of EZH2 in endometrial cancer and evaluated its therapeutic potential.

## RESULTS

### EZH2 is overexpressed in endometrial cancer cell lines and tissues

**Figure 1:**
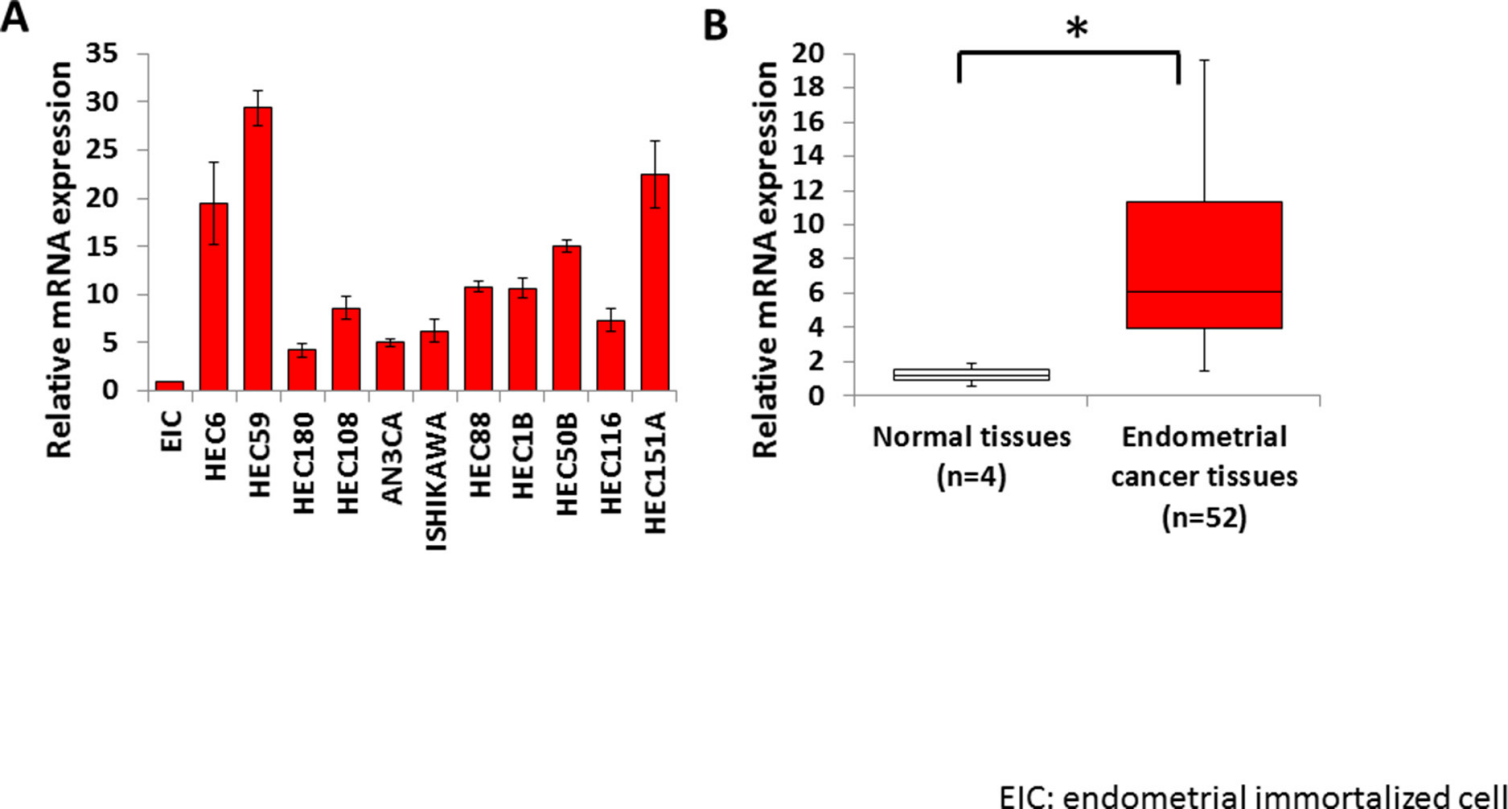
High EZH2 expression in endometrial cancer *EZH2* mRNA levels were measured by qPCR in endometrial cancer cell lines **(A)** and clinical endometrial cancer specimens **(B)**, and compared with those in EICs and normal endometrial tissues, respectively (*P < 0.01). The results show the mean ± SD of three independent experiments.

To identify the histone methyltransferases involved in human endometrial cancer, we examined the expression of several histone methyltransferase genes in 11 endometrial cancer cell lines by real-time qPCR. Significantly elevated expression of *EZH2* was detected in endometrial cancer cell lines compared with that in EICs (non-tumor control) (Figure 1A). *EZH2* expression was significantly higher in the 52 endometrial cancer tissues than in the four normal control tissues (Figure 1B). These results imply that EZH2 is overexpressed in endometrial cancer.

### EZH2 is an independent prognostic factor in endometrial cancer

**Figure 2:**
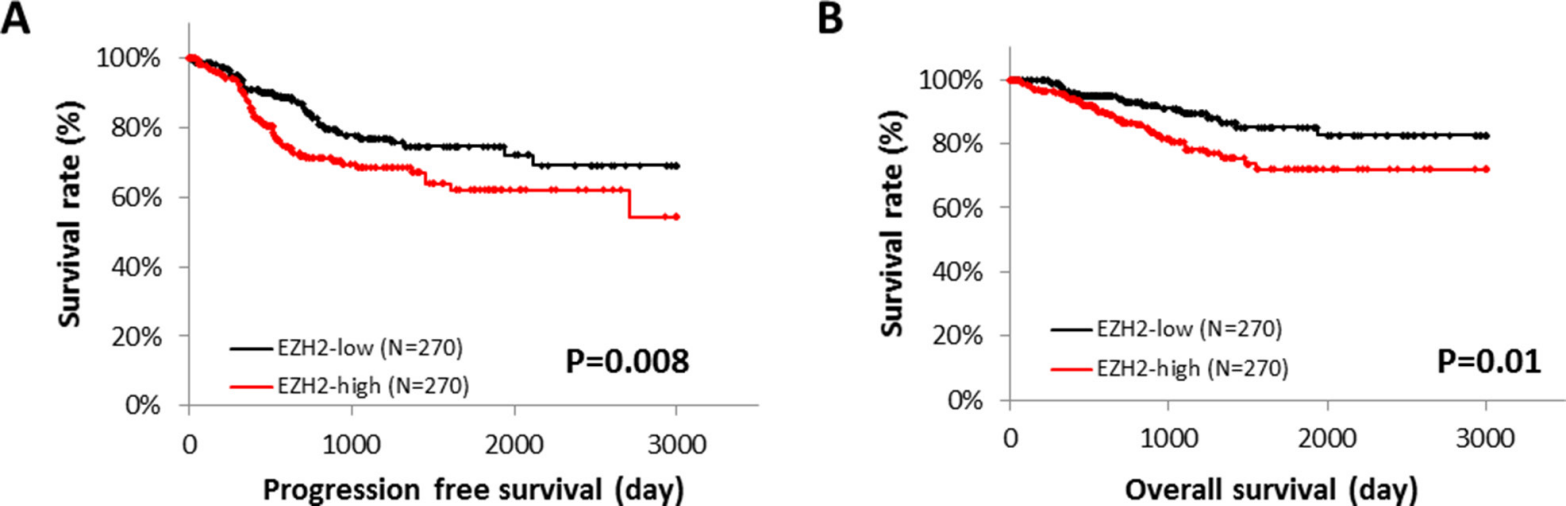
EZH2 expression and patient survival The role of *EZH2* mRNA expression in endometrial cancer was identified by RNA sequencing of 540 cases from the TCGA database. The median of EZH2 expression level was used as the threshold. Progression-free survival (PFS) **(A)** and overall survival (OS) **(B)** were analyzed by the Kaplan-Meier method and log-rank test.

**Figure 3:**
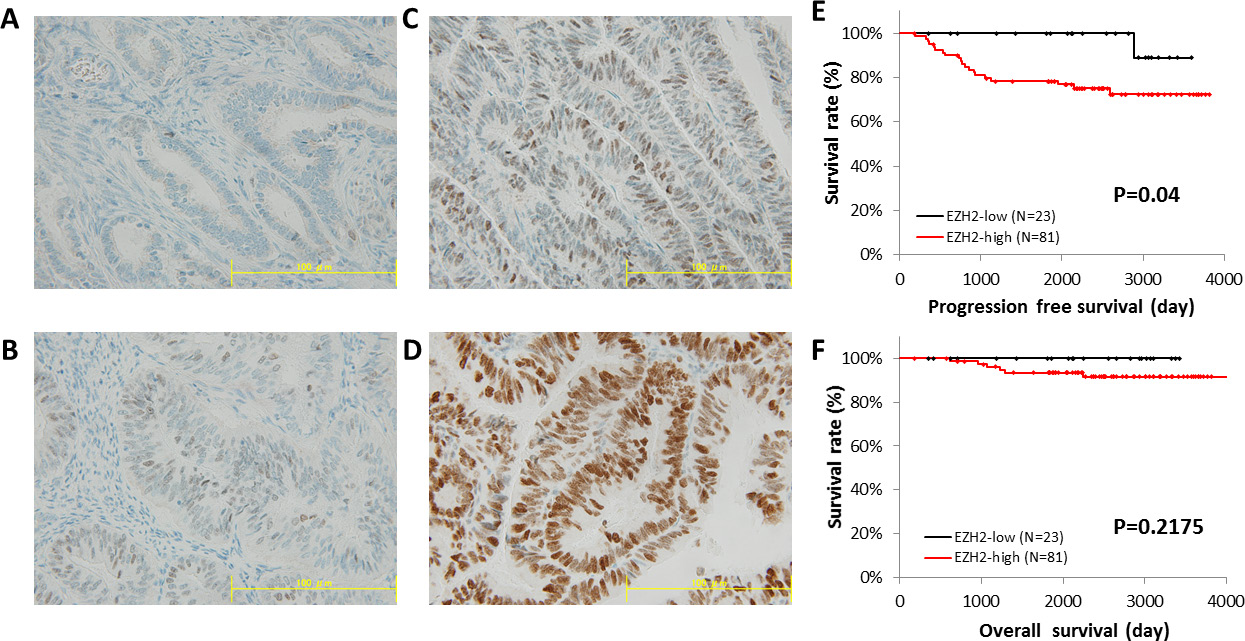
Immunohistochemical staining of EZH2 in a tissue microarray EZH2 protein expression in endometrial cancer was identified using tissue microarray (TMA) data for 104 cases. The intensity of EZH2 expression in TMA was graded as **(A)** 0, negative intensity; **(B)** 1, weak intensity; **(C)** 2, medium intensity; and **(D)** 3, strong intensity. Scale bar, 100 μm. The median intensity of EZH2 expression was used as a threshold. PFS **(E)** and OS **(F)** were analyzed by the Kaplan-Meier method and log-rank test.

Next, we explored the correlation between *EZH2* expression and patient survival, using RNA sequencing data from the TCGA database. Patients with high *EZH2* expression showed significantly poorer progression-free survival (PFS) (*P* = 0.008) and overall survival (OS) (*P* = 0.01) (Figure 2A and 2B). Immunohistochemical analysis of the TMA specimens of endometrial cancers was performed (Figures 3A–3D), and high EZH2 expression was significantly associated with poor PFS (*P* = 0.04) (Figure 3E) but not with OS (*P* = 0.2175) (Figure 3F).

Multivariate analysis identified EZH2 expression as an independent factor for poor prognosis in endometrial cancer with endometrioid histology (HR = 5.31, 95% CI = 1.04–96.9, *P* = 0.0442) in the TMA data set (Supplementary Table 5). However, EZH2 expression was not an independent factor for poor prognosis in the TCGA data set (Supplementary Table 6).

### EZH2 promotes endometrial cancer cell growth through H3K27 trimethylation

**Figure 4:**
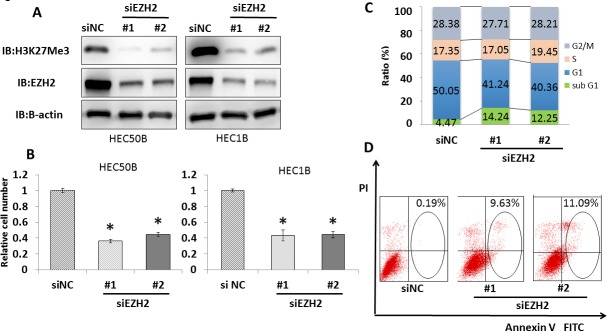
EZH2 knockdown induces significant growth suppression and apoptosis in endometrial cancer cell lines **(A)** EZH2 knockdown decreased EZH2 and H3K27me3 protein levels. After treatment with two different EZH2 siRNAs (siEZH2#1 and siEZH2#2) and control siRNA (siNC) for 48 h in HEC50B and HEC1B, western blotting was performed for EZH2 and H3K27 me3. **(B)** Cell viability assays after treatment with EZH2 siRNAs for 96 h showed significant growth suppression in HEC50B and HEC1B. The mean ± SD values of three independent experiments are shown (* P < 0.01). **(C)** EZH2 knockdown increased the population of cells in sub-G1 phase, as shown by cell cycle analysis. HEC1B cells were treated with siNC and EZH2 siRNAs for 48 h, and cell cycle status was analyzed by flow cytometry and PI staining. **(D)** The proportion of apoptotic cells increased after EZH2 knockdown. HEC1B cells were treated with siNC and EZH2 siRNAs for 48 h, and Annexin V-positive cells were identified by flow cytometry and staining with PI and Annexin V.

To investigate the role of EZH2 in endometrial cancer, we performed knockdown experiments using siRNA against EZH2 (siEZH2#1 and #2) and control siRNA (siNC) in endometrial cancer cell lines. EZH2 knockdown in all examined cell lines was confirmed by immunoblotting. EZH2 knockdown decreased trimethylation levels of histone H3 at lysine 27 (H3K27 me3) (Figure 4A, Supplementary Figure 1A). Significant growth suppression was detected in the four endometrial cancer cell lines after EZH2 knockdown (Figure 4B, Supplementary Figure 1B). To further clarify the mechanism of growth suppression induced by siRNA, we performed flow cytometry analysis. The sub-G1 population of cancer cells was significantly increased by EZH2 knockdown, indicating that EZH2 knockdown induces apoptosis in endometrial cancer cells (Figure 4C). Furthermore, the population of apoptotic cells was also examined by Annexin V-FITC/PI assay. EZH2 knockdown significantly increased the number of apoptotic cells (Figure 4D). Thus, EZH2 knockdown suppresses endometrial cancer cell growth and induces apoptosis.

### GSK126 retards endometrial cancer cell proliferation and increases the number of apoptotic cells

**Figure 5:**
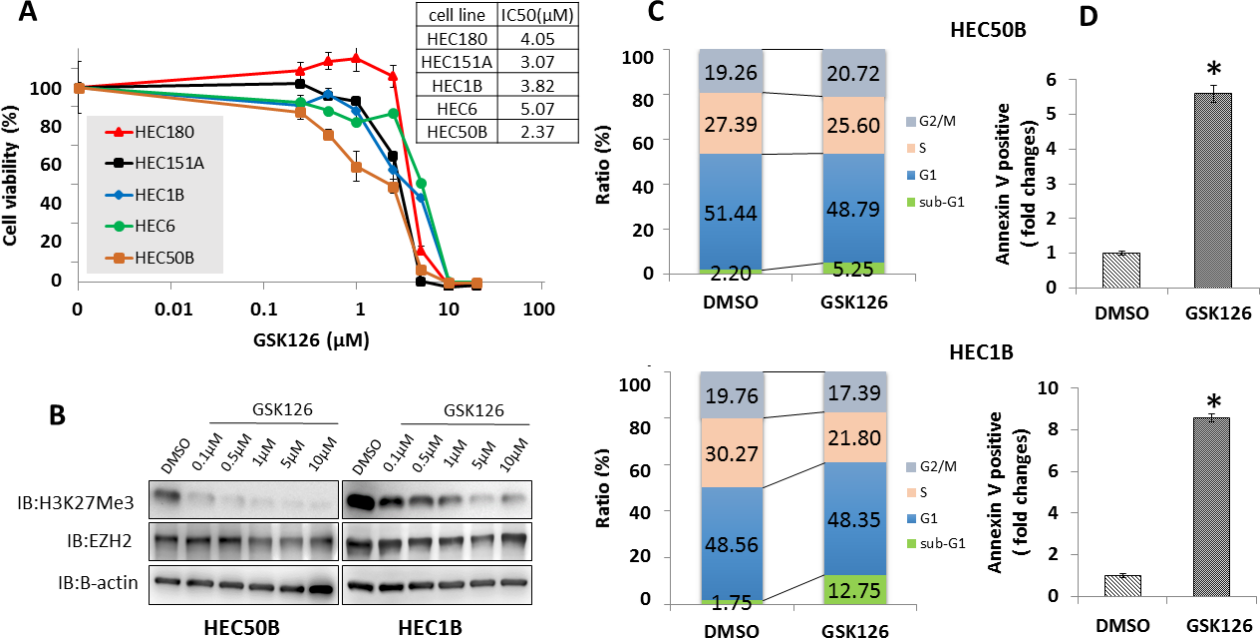
Effect of GSK126 on endometrial cancer cell lines *In vitro* sensitivity of endometrial cancer cell lines to GSK126. **(A)** After treatment with various concentrations of GSK126 (0.025–20 μM) for 8 days, five endometrial cancer cell lines were subjected to cell viability assay. Cell viability (%) was normalized using cells treated with 0.4% dimethyl sulfoxide (DMSO). The IC_50_ values of GSK126 in endometrial cancer cell lines ranged from 2.37–5.07 μM. **(B)** Increasing concentrations of GSK126 consistently decreased H3K27 me3 protein levels. After HEC50B and HEC1B cells were treated with various concentrations of GSK126 (0.1–10 μM) or 0.2% DMSO for 24 h, western blotting was performed for EZH2 and H3K27 me3. **(C)** GSK126 increased the proportion of cells in sub-G1 phase. HEC50B and HEC1B cells were treated with 10 μM GSK126 or 0.2% DMSO for 72 h. Cell cycle status was analyzed by flow cytometry and PI staining. **(D)** GSK126 induced apoptosis. HEC50B and HEC1B cells were treated with 10 μM GSK126 or 0.2% DMSO for 72 h. The proportion of Annexin V-positive cells was calculated by flow cytometry and staining with PI and Annexin V. The results show the mean ± SD of three independent experiments (* P < 0.01).

Treatment with the EZH2 inhibitor GSK126 suppressed the growth of endometrial cancer cell lines, with an increasing effect observed at increasing concentrations (IC_50_: 2.37–5.07 μM) (Figure 5A) and reduced H3K27 me3 levels (Figure 5B). To determine whether the growth-inhibitory effect of GSK126 resulted from cell cycle arrest or cell death, GSK126-treated endometrial cancer cell lines were analyzed by flow cytometry. GSK126 significantly increased the sub-G1 population in endometrial cancer cells. (Figure 5C). Annexin V-FITC/PI assay confirmed that increasing concentrations of GSK126 significantly increased the apoptotic cell population in endometrial cancer cells (Figure 5D).

To investigate the effect of estrogen on EZH2, we evaluated the expression of Erα in the endometrial cancer cell lines we used in this project. There was no significant ERα expression in the five endometrial cancer cell lines tested (Supplementary Figure 3A). In addition, EZH2 expression was not affected by exposure to estrogen in HEC1B and HEC50B cells (Supplementary Figure 3B).

**Figure 6:**
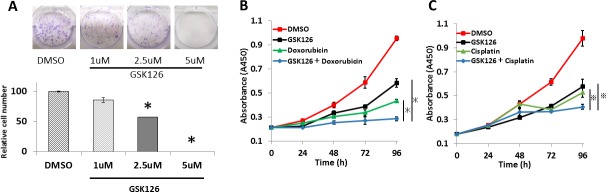
GSK126 suppresses colony formation and shows additive effects with doxorubicin and cisplatin on endometrial cancer cells **(A)** After treatment with various concentrations of GSK126 (1–5 μM) for 9 days, HEC50B colonies were counted and normalized against the number of colonies observed after treatment with 0.2% DMSO. **(B)** HEC1B cells were treated with DMSO, doxorubicin (25 nM), GSK126 (7.5 μM), or both. Cell viability assay was performed at different time points (0–96 h). **(C)** HEC1B cells were treated with DMSO, cisplatin (1 μM), GSK126 (7.5 μM), or both. Cell viability assay was performed at different time points (0–96 h). The results show the mean ± SD of three independent experiments (*: *P*< 0.01, ※: *P* < 0.05).

Colony formation assays showed that increased concentrations of GSK126 decreased the number of colonies formed by endometrial cancer cell lines (Figure 6A). We explored the anti-tumor effects of combinations of GSK126 and key chemotherapeutic agents against endometrial cancer, i.e., cisplatin and doxorubicin, on endometrial cancer cell lines. Combined treatment with GSK126 and doxorubicin or cisplatin additively inhibited cell proliferation. (Figure 6B, 6C and Supplementary Figure 2).

## DISCUSSION

Here, we showed that EZH2 is overexpressed in endometrial cancer cells compared with that in normal cells, and high EZH2 expression is significantly associated with poor prognosis in endometrial cancer. Our results indicate that EZH2 promotes the growth of endometrial cancer cells by increasing H3K27 trimethylation. Further, we confirmed that *EZH2* knockdown suppresses the growth of endometrial cancer cells and induces apoptosis. Finally, we also show that a selective EZH2 inhibitor suppresses the growth of endometrial cancer cells.

EZH2 has been shown to be frequently overexpressed in various types of tumors, and a higher level of EZH2 might be associated with poor prognosis [22]. Dysregulation of EZH2-catalyzed H3K27 trimethylation is frequently observed in many types of cancers. Our study suggests that high EZH2 expression is a significant prognostic factor in endometrial cancer, according to the TCGA and TMA data. These results are congruent with other findings from immunohistochemical experiments [18, 19]. In addition, we showed via quantitative real-time PCR that EZH2 is significantly overexpressed in 11 endometrial cancer cell lines and clinical samples compared with that in normal samples. The oncogenic function of EZH2-catalyzed methylation is known to rely on transcriptional silencing of tumor-associated genes. Additional findings to support an oncogenic role for EZH2 have recently emerged; recurrent mutations of the tyrosine 641 (Y641) and alanine 677 (A677) residues of EZH2 have been reported in B-cell lymphomas [23, 24]. Although the Y641 mutant was initially reported to be a loss-of-function mutation [25], subsequent biochemical analyses indicated that this mutant actually caused increased activity of EZH2 [23, 26]. Similar to the Y641 mutant, the A677 mutation led to aberrantly elevated H3K27 trimethylation [24]. These findings indicate that the role of EZH2 in human tumorigenesis primarily involves dysregulation of H3K27 trimethylation. In this study, *EZH2* knockdown decreased cell proliferation and induced apoptosis by decreasing the H3K27 me3 level in endometrial cancer cell lines. Moreover, GSK126 treatment decreased endometrial cancer cell growth and increased apoptotic cell death by attenuating H3K27 trimethylation. Previous studies have shown that EZH2 knockdown decreases proliferation in endometrial cancer cells [20, 21]. In addition, apoptotic effects of EZH2 inhibition have been reported in other cancers, including lung cancer [27]. However, this is the first report of the effectiveness of EZH2 inhibition in endometrial cancer.

EZH2-specific inhibitors have recently been developed, and their anti-tumor activity has been demonstrated in various cancer types, including B-cell lymphoma [28, 29]. In this study, the IC_50_ of GSK126 in endometrial cancer cells was micromolar, which is similar to that reported in other types of cancers such as prostate cancer [30]. In addition, the present colony formation assays indicated a long-term effect of GSK126 on endometrial cancer cells. Importantly, combination of GSK126 with chemotherapeutic agents such as doxorubicin and cisplatin had an additive effect on endometrial cancer cells.

Randomized trials have reported that doxorubicin and cisplatin chemotherapy improves the PFS and OS of advanced endometrial cancer [31, 32]. These results indicate that combined administration of a small-molecule EZH2 inhibitor such as GSK126 with conventional chemotherapy could be a new strategy for effective treatment of endometrial cancer. Several *in vivo* studies evaluating the use of GSK126 against various types of cancer cells in xenograft mouse models suggest its efficacy and feasibility [30, 33, 34]. In addition, a phase I clinical trial of GSK126 for patients with relapsed-refractory, diffuse large B-cell lymphoma and transformed follicular lymphoma has already started in the United States (ClinicalTrials.gov identifier: NCT02082977). The clinical significance of EZH2 inhibitors such as GSK126 in the treatment of various types of cancer, including endometrial cancer, should be explored further in future studies.

It has been reported that EZH2 is transcriptionally induced by estradiol in ERα-positive breast cancer cell lines [35]. Therefore, a correlation between EZH2 expression and estrogen may exist in ERα-positive endometrial cancer cell lines. However, neither significant ERα expression nor a significant effect of estrogen on EZH2 expression was observed in the endometrial cancer cell lines in this study.

This study has some limitations. First, biomarkers for sensitivity to GSK126 in endometrial cancer cells have not yet been identified. Second, an experiment using a xenograft model is required to clarify the anti-tumor effect of GSK126 against endometrial cancer. Third, although the established function of EZH2 in tumorigenesis is known to require H3K27 trimethylation with transcriptional silencing of tumor-associated genes, several recent studies have shown some non-established functions of EZH2. For example, EZH2 was shown to methylate and competitively inhibit the ubiquitination of H2BK120, which is a lysine residue, in cancer cells [36]. In this regard, we did not perform a functional analysis to determine the other roles of EZH2 in endometrial cancer. Further studies relevant to the non-established roles of EZH2 in endometrial cancer are warranted.

In conclusion, the present findings highlight that EZH2 overexpression is involved in endometrial cancer, and that EZH2 might be a promising candidate for treating endometrial cancer. Mono-chemotherapy of a selective EZH2 inhibitor such as GSK126, or combination chemotherapy using a selective EZH2 inhibitor and one or more conventional anticancer drugs, might be an appropriate strategy to cure high-risk endometrial cancer.

## MATERIALS AND METHODS

### Kaplan–Meier survival analysis

*EZH2* gene expression in endometrial cancer was investigated by Kaplan–Meier analysis of RNA sequencing data downloaded from The Cancer Genome Atlas (TCGA; https://tcga-data.nci.nih.gov/) [37] on May 9, 2016. *EZH2* expression was normalized using RNA-Seq by Expectation Maximization [38]. The 540 clinical endometrial cancer samples available were classified into two groups—EZH2-high and EZH2-low—on the basis of the median of *EZH2* expression as a threshold.

### Tumor samples and RNA extraction

Fifty-six clinical endometrial specimens (52 endometrioid adenocarcinoma, four normal endometrial tissue) were obtained from the University of Tokyo Hospital (Supplementary Table 1). Written informed consent from the patients and approval of the Human Genome, Gene Analysis Research Ethics Committee of the University of Tokyo were obtained. RNA was isolated from the supernatant, using RNeasy Mini Kit (Qiagen, Valencia, CA, USA).

### Reverse transcription and quantitative real-time PCR

For quantitative real-time PCR (qPCR), specific primers for GAPDH (housekeeping gene), and EZH2 were designed (primer sequences in Supplementary Table 2) [22]. cDNA for reverse transcription and qPCR was synthesized from the total RNA of the 56 clinical samples and 12 endometrial cancer cells, using ReverTra Ace-a- (Toyobo, Osaka, Japan). *EZH2* mRNA levels were measured by qPCR, using the One Step SYBR Prime Script RT-PCR Kit (TaKaRa Bio, Tokyo, Japan) in a Light Cycler instrument (Roche), and normalized to the mRNA levels of *GAPDH*.

### Tissue microarray

In total, 104 clinical endometrial cancer specimens were obtained from the University of Tokyo Hospital (Supplementary Table 3). Written informed consent was obtained from the patients. The Human Genome, Gene Analysis Research Ethics Committee of the University of Tokyo approved the study design. All of the patients showed endometrioid histology. Formalin-fixed, paraffin-embedded primary endometrial cancer specimens were cut into 4-μm-thick sections for tissue microarray (TMA).

### Immunohistochemical staining

EZH2 expression patterns in the TMAs of endometrial cancer specimens were analyzed by immunohistochemical staining (Ventana Benchmark XT autostainer; Ventana Medical Systems Inc., Tucson, AZ, USA) [39].

Immunohistochemical analysis was performed using an anti-EZH2 antibody (Cell Signaling Technology, 5246). EZH2 expression intensity was graded from 0 to 3. The results were scored independently by two observers, and the average score was recorded as the final expression score for evaluation.

### Cell culture

AN3CA and HEC1B endometrial cancer cell lines and the MCF-7 breast cancer cell line were purchased from the American Type Culture Collection (Manassas, VA, USA). Ishikawa and endometrial immortalized cells (EICs) were generous gifts from Dr. Masato Nishida (Kasumigaura Medical Centre, Japan) and Dr. Satoru Kyo (Shimane University, Japan) [40], respectively. The eight other cell lines were established by Dr. Hiroyuki Kuramoto (Kanagawa Health Service Association, Japan). All cell lines were cultured in Eagle's minimal essential medium (MEM) with 10% fetal bovine serum (FBS) and antibiotics at 37°C in a humidified incubator with 5% CO_2_.

### Gene silencing

siRNA oligonucleotide duplexes designed to downregulate EZH2 and the negative control siRNA (siNC; MISSION siRNA Universal Negative Control) were obtained from Sigma-Aldrich (Supplementary Table 4). Cells (2 × 10^5^/well) were seeded in 6-well plates for western blotting, cell cycle analysis, and Annexin V-FITC/PI assay. Another set of cells (1 × 10^4^/well) was seeded in 24-well plates for cell viability assay. After 24 h of incubation, siRNA duplexes (100 nM) were transfected with Lipofectamine-RNAi MAX transfection reagent (Invitrogen, Carlsbad, CA, USA) for 48–96 h before analysis.

### Cell viability assay

Cells (500 cells/well) were seeded in 24-well plates with MEM. After 24 h, the medium was replaced with fresh medium containing various concentrations of GSK126 (Active Biochemicals, Maplewood, NJ, USA), cisplatin (Nichi-Iko Pharmaceutical, Toyama, Japan), and doxorubicin (Sigma-Aldrich, St. Louis, MO, USA). Cell Counting Kit-8 solution (50 μL; Dojindo, Tokyo, Japan) was added to each well before incubation for 2 h. Cell viability was normalized using cells treated with 0.4% dimethyl sulfoxide (DMSO).

### Cell cycle analysis

Cells (2×10^5^/well) were seeded in 6-well plates. After 24h of incubation, the medium was replaced with fresh medium containing various concentrations of GSK126 or the siRNAs, and incubated for 72 h. Cells were collected using trypsin and stained in the dark with 50 μg/mL propidium iodide (PI) (Sigma-Aldrich, St. Louis, MO, USA) at 4°C for 30 min. Cell cycle distribution was analyzed by flow cytometry on an Epics XL instrument (Beckman Coulter, Brea, CA, USA) using Cell Quest Pro software v 3.1 (BD Bioscience, Franklin Lakes, NJ, USA).

### Detection of apoptosis

Cells (2×10^5^/well) were seeded in 6-well plates. After 24 h of incubation, the medium was replaced with fresh medium containing GSK126 or the siRNAs and incubated for 24–72 h. Cells were collected using trypsin and washed twice with phosphate-buffered saline (PBS). Collected cells were re-suspended in 1× binding buffer and stained with fluorescein isothiocyanate-conjugated (FITC) Annexin V/PI (Annexin V-FITC Apoptosis Detection kit II; BD Bioscience) in the dark at room temperature for 15 min. Annexin V-FITC/PI double-positive cells were detected by flow cytometry and expressed as a percentage of apoptotic cells.

### Colony formation assay

Cells (1 × 10^3^/well) were seeded in 6-well plates. After 24 h of incubation, the medium was replaced with fresh medium containing various concentrations of GSK126 or DMSO, followed by 9 days of incubation. The medium and GSK126 were replaced every 3 days. The cells were fixed with 100% methanol for 2 h and stained with Giemsa (Wako, Osaka, Japan). Colonies having more than 100 cells were counted and normalized to the number of colonies observed after treatment with 0.2% DMSO [41].

### Western blotting

Total protein was transferred to nitrocellulose membranes. The membranes were probed with an anti-EZH2 antibody (Leica Biosystems, PA0575), anti-H3K27 me3 antibody (Cell Signaling Technology, 9733), anti-estrogen receptor α antibody (Cell Signaling Technology, 8644) and anti-ACTB antibody (Sigma-Aldrich, A2228). Protein bands were detected with the Enhanced Chemiluminescence Select Western Blotting detection kit (GE Healthcare Life Sciences, Piscataway, NJ, USA).

### Estrogen exposure

After treatment of HEC1B and HEC50B with 10 nM E2 (Sigma-Aldrich, St. Louis, MO, USA) or reduced-serum medium (Life Technologies Japan Ltd, Tokyo, Japan) for 90 min or 180 min, western blotting was performed for EZH2 and ERα.

### Statistical analysis

P < 0.05 was considered significant. Survival curves were constructed by the Kaplan–Meier method and log-rank test. For the other experiments, statistical significance was determined by Student's *t*-test, using bell curves in MS Excel (Social Survey Research Information, Japan) and JMP Pro 12 (SAS, Cary, NC, USA). Each experiment was repeated thrice, and mean ± standard deviation values were calculated.

## SUPPLEMENTARY MATERIALS FIGURES AND TABLES






