# Supplementary material for: Oncogenic histone methyltransferase EZH2: A novel prognostic marker with therapeutic potential in endometrial cancer
Source: Oncotarget. 2017 Mar 17;8(25):40402–11. doi: 10.18632/oncotarget.16316 (PMC5522273; doi:10.18632/oncotarget.16316)
Supplement: Supplementary file 1 [file oncotarget-08-40402-s001.pdf]

Oncogenic histone methyltransferase EZH2: A novel prognostic marker with therapeutic potential in endometrial cancer

SUPPLEMENTARY MATERIALS

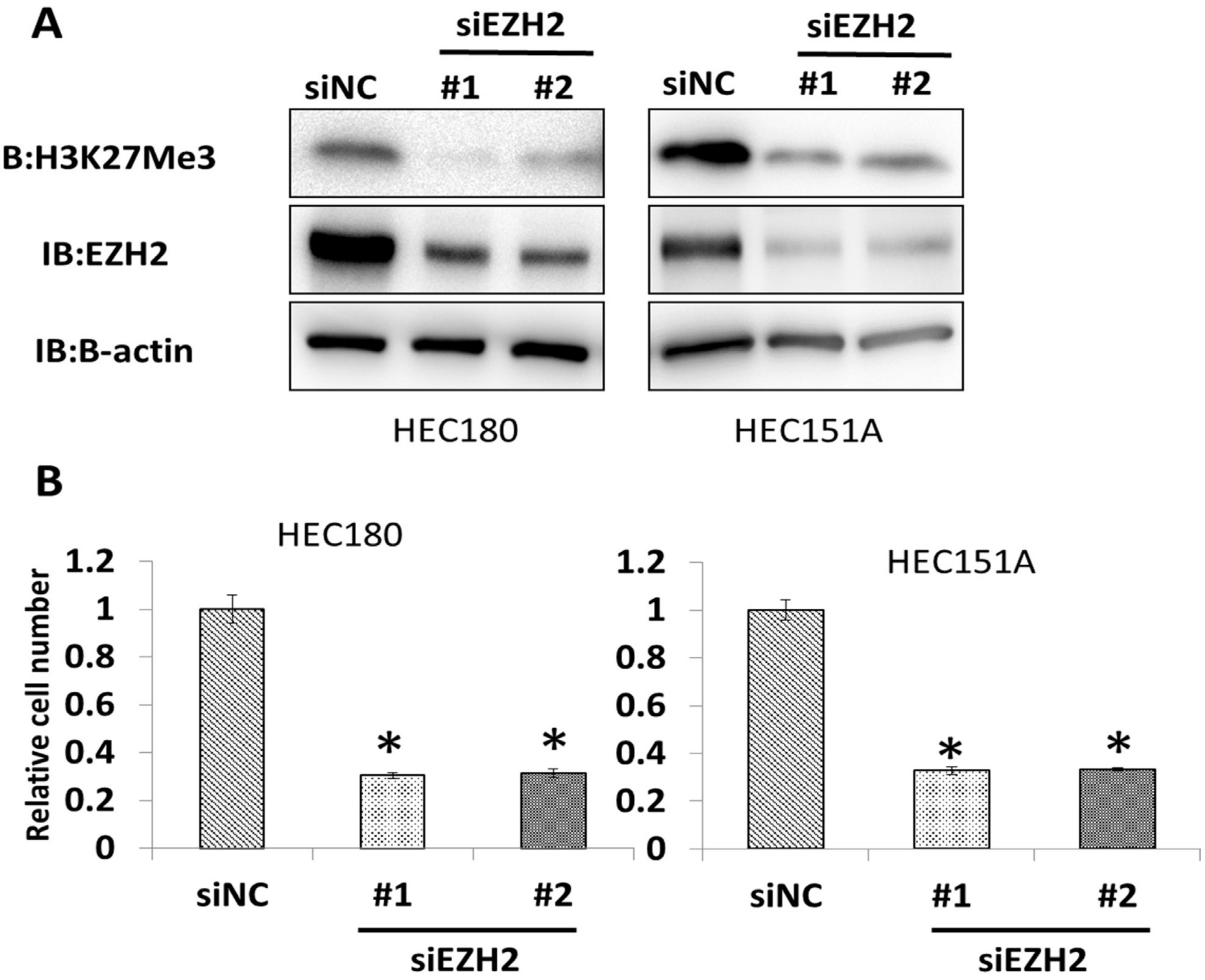

Supplementary Figure 1: Knockdown of EZH2 induces significant growth suppression in endometrial cancer cell lines. **(A)** EZH2 knockdown decreased EZH2 and H3K27me3 protein levels. After treatment of 2 different EZH2 siRNAs (siEZH2#1 and siEZH2#2) and control siRNA (siNC) for 48h in HEC151A and HEC180, western blotting was performed for EZH2 and H3K27me3. **(B)** EZH2 knockdown induced significant growth suppression. Cell viability assays were performed using Cell Counting Kit-8 (Dojindo) after treatment of EZH2 siRNAs for 96h in HEC151A and HEC180.

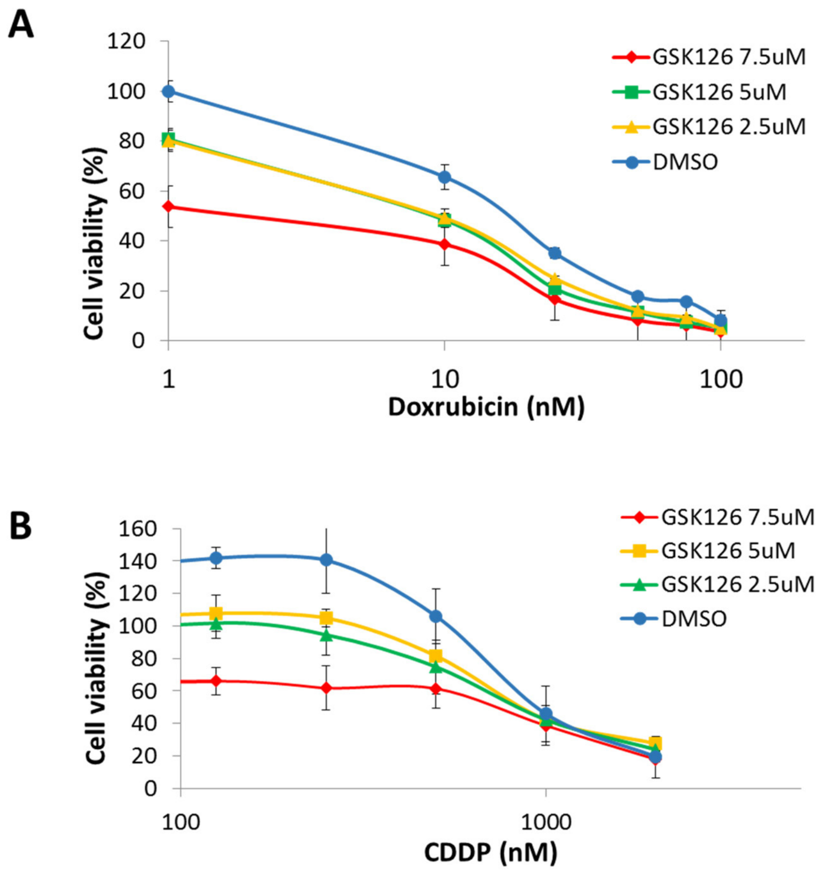

Supplementary Figure 2: Combination of GSK126 with doxorubicin and cisplatin had an additive effect on endometrial cancer cells. (A) *In vitro* sensitivity of endometrial cancer cell lines to the combination of GSK126 and doxorubicin. After treatment of HEC1B cells with various concentrations of GSK126 (2.5-7.5 µM) and doxorubicin (1-100 nM) for 5 days, a cell viability assay was performed. Cell viability (%) was normalized to that of cells treated with 0.15% DMSO. **(B)** After treatment of HEC1B cells with various concentrations of GSK126 (2.5-7.5 µM) and cisplatin (125-2000 nM) for 5 days, a cell viability assay was performed. Cell viability (%) was normalized to that of cells treated with 0.15% DMSO.

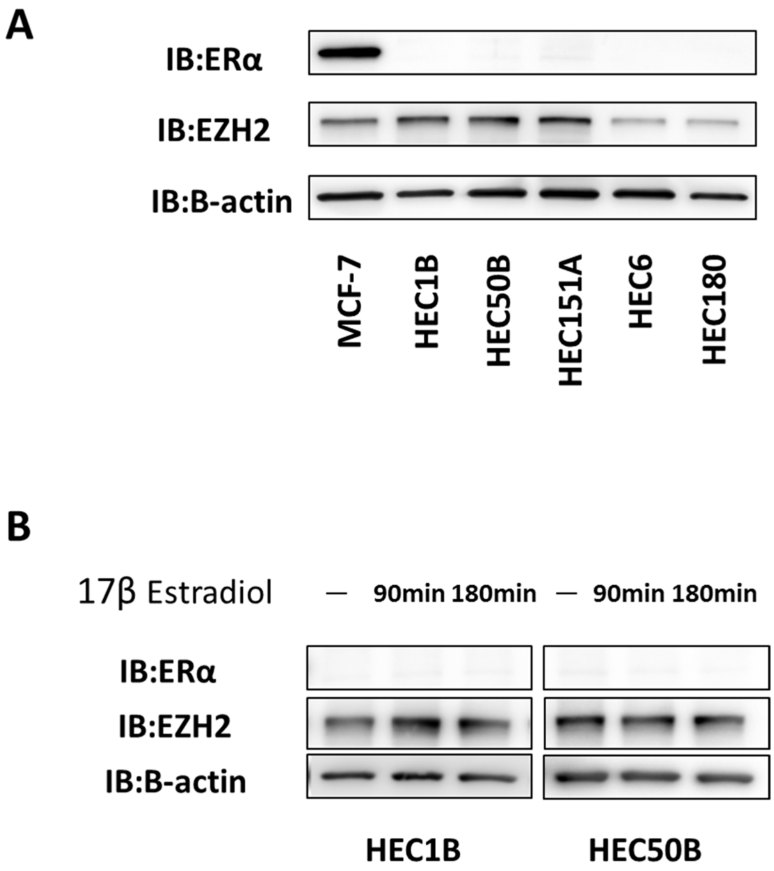

Supplementary Figure 3: Effect of 17β-estradiol (E2) on EZH2 in endometrial cancer cell lines. (A) The protein levels of estrogen receptor α (ERα) in endometrial cancer cell lines. **(B)** ERα-positive MCF-7 (breast cancer cell lines) were measured by western blotting. **C.** After treatment of HEC1B and HEC50B with 10 nM E2 or reduced-serum medium for 90 min or 180 min, western blotting was performed for EZH2 and ERα.

Supplementary Table 1: Clinicopathological background in 52 patients with endometrial cancer

See Supplementary File 1

Supplementary Table 2: Primer Sequences for Quantitive RT-PCR

Gene Name	Primer Sequence
GAPDH (housekeeping gene)-f	5′-GAAGGTGAAGGTCGGAGTC-3′
GAPDH (housekeeping gene)-r	5′-GAAGATGGTGATGGGATTTC-3′
EZH2-f	5′-CGCTTTTCTGTAGGCGATGT-3′
EZH2-r	5′-TGGGTGTTGCATGAAAAGAA-3′

Supplementary Table 3: Clinicopathologic characteristics of endometrial cancer tissues on the Tissue Microarray

See Supplementary File 2

Supplementary Table 4: siRNA Sequences

siRNA Name	Sequence	
siEZH2 #1	Sense:	5′-CUAACCAUGUUUACAACUA-3′
	Antisense:	5′-UAGUUGUAAACAUGGUUAG-3′
siEZH2 #2	Sense:	5′-GACAGAAGAGGGAAAGUGU-3′
	Antisense:	5′-ACACUUUCCCUCUUCUGUC-3′

Supplementary Table 5: Univariate/Multivariate analysis of EZH2 expression in endometrial cancers, using TMA

Characteristics	Univariate analysis			Multivariate analysis		
	HR	95% CI	P-value	HR	95% CI	P-value
Stage						
Advanced (III/IV)	1.57	0.62-3.74	0.3229	1.29	0.44-3.56	0.6327
Early (I/II)						
Grade						
G3	1.16	0.34-7.25	0.8429			
G1/G2						
Age						
≥60	1.57	0.65-3.75	0.304	1.36	0.55-3.31	0.4996
<60						
Muscle invasion						
>1/2	0.56	0.09-1.95	0.407	0.37	0.06-1.35	0.1459
≤1/2						
EZH2 expression						
Strong	6.25	1.30-112.2	0.017	5.31	1.04-96.9	0.0442
Weak						
Lymphatic invasion						
Yes	1.8	0.68-4.33	0.2222	1.17	0.38-3.47	0.7774
No						
Vascular invasion						
Yes	2.59	1.02-6.15	0.0448	2.41	0.89-6.13	0.0818
No						

Supplementary Table 6: Univariate/Multivariate analysis of EZH2 expression in 540 endometrial cancers, using RNA sequencing data from TCGA

Characteristics	Univariate analysis			Multivariate analysis		
	HR	95% CI	P-value	HR	95% CI	P-value
Stage						
Advanced (III/IV)	2.84	1.97-4.09	<0.0001	2.95	1.89-4.58	<0.0001
Early (I/II)						
Grade						
G3	1.78	1.20-2.71	0.042	1.13	0.66-1.94	0.665
G1/G2						
Age						
≥60	1.56	1.05-2.41	0.0288	1.43	0.88-2.39	0.1559
<60						
Muscle invasion						
>1/2	2.47	1.66-3.67	<0.0001	1.68	1.08-2.60	0.0201
≤1/2						
EZH2 expression						
High	1.63	1.08-2.55	0.0187	1.35	0.82-2.32	0.2444
Low						
Histology						
Non-endometrioid	1.87	1.28-2.71	0.0015	1.19	0.71-2.03	0.5097
Endometrioid						
