# Supplementary material for: Oncogenic histone methyltransferase EZH2: A novel prognostic marker with therapeutic potential in endometrial cancer
Source: Oncotarget. 2017 Mar 17;8(25):40402–11. doi: 10.18632/oncotarget.16316 (PMC5522273; doi:10.18632/oncotarget.16316)
Supplement: Supplementary file 2 [file oncotarget-08-40402-s002.pdf]

Table 1. Clinicopathological background in 52 patients with endometrial cancer.

Case No.	Age (years)	Histologic Diagnosis	Grade	Stage (TNM)
1	47	Normal	-	-
2	31	Normal	-	-
3	41	Normal	-	-
4	unclear	Normal	-	-
5	42	Endometroid adenocarcinoma	G1	pT1aN0M0
6	58	Endometroid adenocarcinoma	G1	pT1aN0M0
7	51	Endometroid adenocarcinoma	G1	pT1aN0M0
8	34	Endometroid adenocarcinoma	G1	pT1aN0M0
9	39	Endometroid adenocarcinoma	G1	pT1aN0M0
10	31	Endometroid adenocarcinoma	G1	pT1aN0M0
11	66	Endometroid adenocarcinoma	G1	pT1aN0M0
12	37	Endometroid adenocarcinoma	G2	pT1aN0M0
13	40	Endometroid adenocarcinoma	G2	pT1aN0M0
14	65	Endometroid adenocarcinoma	G2	pT1aN0M0
15	52	Endometroid adenocarcinoma	G2	pT1aN0M0
16	46	Endometroid adenocarcinoma	G2	pT1aN0M0
17	60	Endometroid adenocarcinoma	G3	pT1aN0M0
18	43	Endometroid adenocarcinoma with squamous differentiation	G3	pT1aN0M0
19	31	Endometroid adenocarcinoma with squamous differentiation	G3	pT1aN0M0
20	60	Endometroid adenocarcinoma	G1	pT1bN0M0
21	73	Endometroid adenocarcinoma	G2	pT1bN0M0
22	67	Endometroid adenocarcinoma	G2	pT1bN0M0
23	64	Endometroid adenocarcinoma	G2	pT1bN0M0
24	68	Endometroid adenocarcinoma	G2	pT1bN0M0
25	55	Endometroid adenocarcinoma	G3	pT1bN0M0
26	51	Endometroid adenocarcinoma	G3	pT1bN0M0
27	54	Endometroid adenocarcinoma	G3	pT1bN0M0
28	79	Endometroid adenocarcinoma	G3	pT1bNXM0
29	52	Endometroid adenocarcinoma	G3	pT1bN0M0
30	57	Endometroid adenocarcinoma	G1	pT2N0M0
31	48	Endometroid adenocarcinoma	G1	pT2N0M0
32	44	Endometroid adenocarcinoma with squamous differentiation	G1	pT2N0M0
33	72	Endometroid adenocarcinoma	G1	pT2N0M0

34	68	Endometroid adenocarcinoma with squamous differentiation	G2	pT2N0M0
35	48	Endometroid adenocarcinoma with squamous differentiation	G2	pT2N0M0
36	42	Endometroid adenocarcinoma	G3	pT2N0M0
37	66	Endometroid adenocarcinoma with squamous differentiation	G1	pT3bN0M0
38	66	Endometroid adenocarcinoma	G3	pT3bN0M0
39	45	Endometroid adenocarcinoma with squamous differentiation	G1	pT2N1M0
40	57	Endometroid adenocarcinoma	G1	pT1aN1M0
41	63	Endometroid adenocarcinoma with squamous differentiation	G2	pT3bN1M0
42	53	Endometroid adenocarcinoma with squamous differentiation	G1	pT1bN1M0
43	59	Endometroid adenocarcinoma	G1	pT1bN1M0
44	66	Endometroid adenocarcinoma	G1	pT2N1M0
45	63	Endometroid adenocarcinoma	G1	pT2N1M0
46	75	Endometroid adenocarcinoma	G2	pT3aN1M0
47	71	Endometroid adenocarcinoma	G2	pT3bN1M0
48	56	Endometroid adenocarcinoma	G3	pT1bN1M0
49	48	Endometroid adenocarcinoma	G3	pT1bN1M0
50	66	Endometroid adenocarcinoma	G3	pT3aN1M0
51	41	Endometroid adenocarcinoma	G3	pT1aN1M0
52	52	Endometroid adenocarcinoma	G3	pTbN1M0
53	44	Endometroid adenocarcinoma	G3	pT3aN1M0
54	64	Endometroid adenocarcinoma	G1	pT3aNXM1
55	45	Endometroid adenocarcinoma	G1	pT3bNXM1
56	44	Endometroid adenocarcinoma	G3	pT4N1M0
